# Are the Soluble Receptors sRAGE, sRANKL, and Osteoprotegerin Associated with Anemia in Rheumatoid Arthritis?

**DOI:** 10.3390/ijms252312729

**Published:** 2024-11-27

**Authors:** Katya Stefanova, Ginka Delcheva, Teodora Stankova

**Affiliations:** Department of Medical Biochemistry, Faculty of Pharmacy, Medical University of Plovdiv, 15A Vasil Aprilov Blvd., 4002 Plovdiv, Bulgaria; ginka.delcheva@mu-plovdiv.bg (G.D.); teodora.stankova@mu-plovdiv.bg (T.S.)

**Keywords:** anemia of chronic disease (ACD), iron deficiency anemia (IDA), sRAGE, sRANKL, OPG, rheumatoid arthritis, sTfR-F index

## Abstract

Rheumatoid arthritis (RA) is an inflammatory autoimmune disease with articular and systemic manifestations, and one of the most common is anemia. This study aims to investigate whether the levels of the soluble receptors sRAGE, sRANKL, and OPG are affected by the distribution of RA patients in subgroups according to soluble transferrin receptor/log ferritin (sTfR-F index) and hemoglobin (Hb) levels and to examine their correlation with indicators of iron metabolism, disease activity, and autoimmune and inflammatory changes. The levels of sRANKL and sRAGE were significantly higher in the subgroup of anemia of chronic disease combined with iron deficiency anemia (ACD/IDA) compared to the ACD group: *p* < 0.0001 and *p* < 0.0001. The level of OPG tended to decrease in ACD/IDA (*p* = 0.053). sRAGE was positively correlated with prohepcidin, RF and anti-CCP antibodies, sRANKL, CRP, and IL-6 only in the ACD group. A negative correlation was found between sRAGE, sRANKL, and serum iron only in the ACD/IDA group. sRANKL was positively correlated with OPG, prohepcidin, CRP, IL-6, RF, anti-CCP antibodies, and DAS28 only in the ACD group. Positive correlations were observed between OPG and ferritin, sTfR, CRP, IL-6, RF, and DAS28, and a negative correlation was observed with serum iron only in the ACD group. Therefore, the investigated soluble receptors may serve as reliable biomarkers involved in the pathogenesis of RA and may contribute to the identification of patients at risk of developing combined anemia.

## 1. Introduction

Rheumatoid arthritis (RA) is a chronic systemic inflammatory disease of autoimmune nature with a worldwide prevalence ranging from 0.3 to 1%. RA is characterized by synovial inflammation, which further leads to cartilage damage and bone erosion [1]. The pathophysiology of RA is associated with alterations in the cytokine network (mainly interleukin 6), a large increase in acute-phase reactants (including serum C-reactive protein, hepcidin, and ferritin), and certain autoantibodies (rheumatoid factor and anti-cyclic citrullinated peptide antibodies) [2,3].

RA primarily affects joint function but may also have numerous systemic effects. Anemia is one of the most common extra-articular manifestations in RA, affecting between 30 and 70% of patients [4,5,6]. According to the most recent epidemiological data, the incidence of anemia in the RA population can be even higher, reaching 75.28%. Furthermore, anemia in elderly patients with high RA activity is closely related to the course of RA [7]. There is evidence that RA patients with anemia have a more severe form of the disease and more serious joint damage compared to non-anemic patients [4,8]. In RA, there may be anemia of chronic disease (ACD), iron deficiency anemia (IDA), or a combination of both (ACD/IDA). Anemia of chronic disease in RA is attributed to the cytokine-mediated inhibition of iron utilization, apoptosis of erythroid progenitor cells, and decreased erythropoietin production [3]. Iron deficiency anemia is also frequent in RA and is usually caused by gastrointestinal bleeding due to long-term use of non-steroidal anti-inflammatory drugs (NSAIDs). Functional iron deficiency may also be observed in anemia of chronic disease, caused by overexpression of hepcidin (the central regulator of iron homeostasis). This promotes intracellular storage of iron, thus decreasing circulating iron levels and leading to iron-restricted erythropoiesis [9].

The determination of the soluble transferrin receptor/log ferritin ratio (sTfR-F index) provides a significant advantage in diagnosing the type of anemia [10]. The use of the sTfR-F index allows the accurate distribution of patients as having ACD or IDA and can effectively discriminate ACD from combined ACD/IDA in patients with RA [11,12]. Anemia of chronic disease is a common complication of RA with a negative impact on RA symptoms and is associated with reduced quality of life [13]. Several previous studies demonstrated that anemia may predict radiographic damage in RA [6,14,15].

There is evidence that hemoglobin level in RA patients is associated with disease activity and severity, structural damage of joints, and the presence of certain comorbidities [6,16]. Another study has reported that anemia, defined as hemoglobin (Hb) levels < 13 g/dL in men and <12 g/dL in women, is present in more than a third of RA patients and in a quarter of patients within the first year of the disease. In addition, it has been found that patients with anemia also have more severe physical disability [17].

The anemia in RA reflects additional pathological aspects that cannot be measured by traditional markers of disease activity. Therefore, we were interested in understanding whether the type of anemia affects novel specific and reliable biomarkers—the soluble receptors involved in the pathogenesis of the disease.

The role of osteoclasts in the process of bone loss in RA and the factors responsible for regulating the differentiation and activation of osteoclasts are well known. The receptor activator of nuclear factor-kappa B ligand (RANKL), a member of the tumor necrosis factor superfamily, exists in a soluble form (sRANKL), and by binding to its signaling receptor activator of NF-κB (RANK), stimulates osteoclastogenesis in RA [18,19,20]. By contrast, osteoprotegerin (OPG), a soluble TNF receptor-like molecule, is the naturally occurring inhibitor of RANKL. OPG acts as a decoy soluble receptor, competitively binds to RANKL, and blocks the interaction of RANKL with RANK, thus preventing bone resorption [21,22,23]. It is known that dysregulation of the RANKL/OPG system has been implicated in the pathophysiology of bone remodeling in RA, but the relationship with the changes in iron homeostasis and the type of anemia remains unclear.

The receptor for advanced glycation end products (RAGE) is a multi-ligand member of the immunoglobulin superfamily with a diverse class of ligands that are inducers of inflammatory response [24]. RAGE is expressed by many of the cells that participate in the development of RA, including macrophages, neutrophils, T cells, and synovial fibroblasts [25,26]. Soluble RAGE (sRAGE) is a truncated form of the receptor that keeps the ligand binding site but loses the cytosolic and transmembrane domains [27]. Literature has emerged that offers contradictory findings about the function of sRAGE. A widely held view is that sRAGE acts as an anti-inflammatory molecule as a decoy receptor, blocking RAGE ligand interaction with membrane-bound RAGE [28,29,30]. sRAGE may also exert pro-inflammatory activity that is mediated via the Mac-1/NF-κB pathway in an animal model and in vitro [31]. sRAGE directly causes inflammation by recruiting monocytes and neutrophils and monocyte survival and differentiation to macrophages [32]. These contrasting data suggest that the role of sRAGE in RA pathology deserves further examination.

Previously, our group reported that sRAGE, sRANKL, and OPG are associated with iron metabolism, disease activity, and autoimmune status in RA [33,34]. There are insufficient studies in the literature as to whether the type of anemia and the changes in iron homeostasis are associated with bone metabolism controlled by OPG, RANKL, and RAGE. We were interested in investigating whether the levels of the soluble receptors sRANKL, sRAGE, and OPG are affected by the distribution of RA patients in subgroups according to sTfR-F index and hemoglobin levels and in examining their correlation with indicators of iron metabolism, disease activity, and autoimmune and inflammatory changes in these subgroups. Such a study would provide additional information for predicting bone disorders in states of anemia of inflammation and the combined type of anemia in RA.

## 2. Results

**Figure 1:**
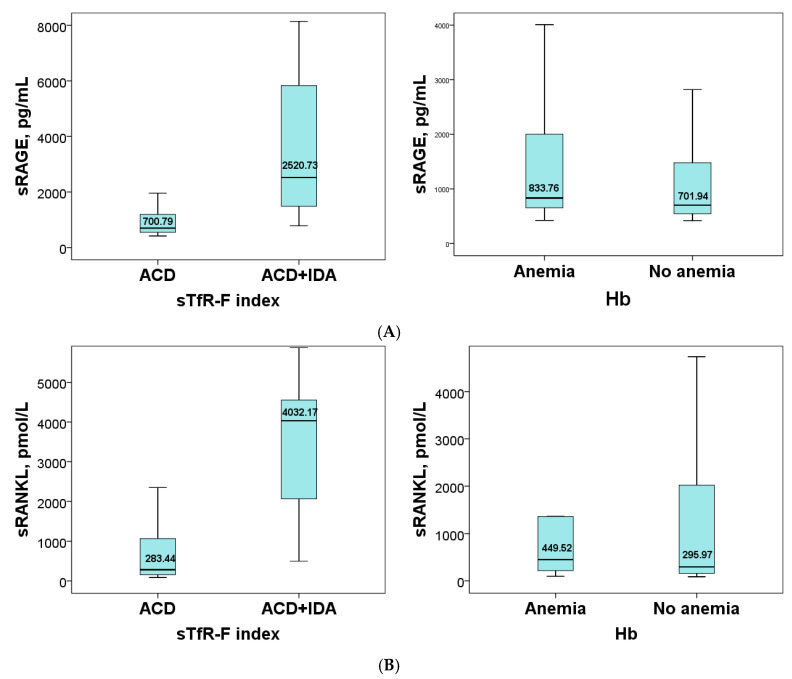

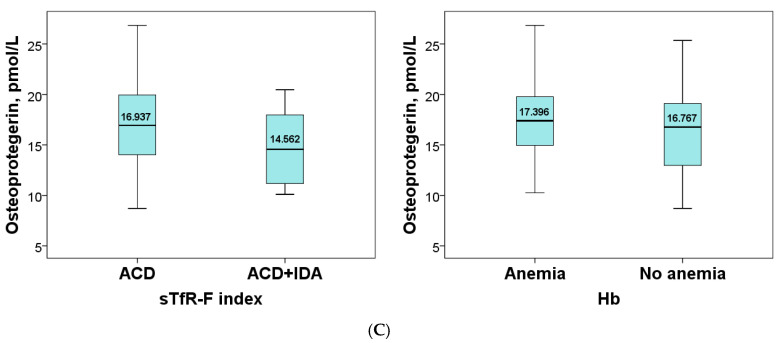
Comparison of sRAGE (**A**), sRANKL (**B**), and OPG (**C**) in subgroups of RA patients according to the level of the sTfR-F index (ACD and ACD + IDA) and Hb (no anemia and anemia).

The serum levels of the soluble receptors (sRAGE, sRANKL, and OPG) according to sTfR-F index value and the level of hemoglobin are shown in Figure 1. The comparison of the soluble receptor content according to the sTfR-F index showed that the levels of sRAGE and sRANKL were significantly higher in the subgroup with combined anemia (ACD/IDA) compared to the ACD subgroup: *p* < 0.0001 and *p* < 0.0001. The level of OPG tended to decrease in ACD/IDA (*p* = 0.053). The distribution of patients according to Hb level did not result in statistically significant differences in sRAGE, sRANKL, and OPG levels between the two subgroups (Figure 1A–C).

Our study included patients on glucocorticoid therapy, but they were equally distributed between the compared subgroups. Since it is known that glucocorticoids could influence the levels of RANKL and OPG, we performed additional statistical analysis excluding all the patients on mono- or combination therapy with glucocorticoids. There were no differences in the observed results before and after the exclusion, and the p-values were almost the same. The statistical significance when comparing the levels of sRAGE, sRANKL, and OPG between the ACD (n = 85) and ACD + IDA (n = 10) subgroups after excluding patients on glucocorticoids were *p* = 0.001, *p* < 0.0001, and *p* = 0.218, respectively. The distribution of patients according to Hb level, after the exclusion of the participants on glucocorticoids, again did not result in any statistically significant difference in the soluble receptor levels between the anemia (n = 27) and no anemia (n = 68) subgroups (*p* > 0.05).

A significant positive correlation was observed between sRAGE and sTfR, regardless of the sTfR-F index value, and in the subgroup with normal Hb levels. sRAGE was positively correlated with prohepcidin and the indicators for the assessment of autoimmune status (RF and anti-CCP antibodies), bone resorption (sRANKL), and inflammation (CRP and IL-6) only in the ACD subgroup (sTfR-F index < 1.5). A negative correlation was found between sRAGE and serum iron, and a positive correlation was found between sRAGE and DAS28 only in the ACD/IDA subgroup (sTfR-F index > 1.5). A positive correlation was observed between sRAGE and sRANKL, regardless of Hb level. There were significant positive correlations between sRAGE and CRP, RF, and anti-CCP antibodies in the subgroup without anemia (with normal Hb level) and with IL-6 in the subgroup with anemia (with low Hb level) (Table 1).

sRANKL was positively correlated not only with sRAGE but with prohepcidin, CRP, IL-6, RF, anti-CCP antibodies, and DAS28 in the ACD subgroup but not in the subgroup with combined anemia (ACD/IDA). In patients with ACD, a significant positive correlation was found between sRANKL and OPG, which was not present in the entire group of RA patients [33]. sRANKL was positively correlated with sTfR, regardless of the type of anemia, as determined by the sTfR-F index. A negative correlation was found between sRANKL and serum iron, and a positive correlation was found between sRANKL and ferritin only in the ACD/IDA subgroup (Table 2).

Positive correlations of sRANKL were found not only with sRAGE and sTfR but also with the CRP, RF, and anti-CCP antibodies, regardless of the Hb level in patients. A significant positive association between sRANKL and prohepcidin was observed only in the subgroup with anemia (low Hb level) (Table 2).

Significant positive correlations were observed between OPG and ferritin, sTfR, sRANKL, CRP, IL-6, RF, and DAS28, and a negative correlation was found with serum iron in the ACD subgroup but not in the combined anemia ACD/IDA subgroup. Significant positive correlations were found for OPG with IL-6 and RF in the anemia subgroup, while in the subgroup without anemia, OPG showed a negative correlation with serum iron and a positive correlation with ferritin (Table 3).

## 3. Discussion

In our previous work, we reported a significantly elevated serum level of sRAGE in patients with RA compared to healthy controls [33]. The results of the present study indicate that sRAGE is a marker sensitive to the distribution of RA patients depending on the type of anemia, assessed by the sTfR-F index. Serum levels of sRAGE were significantly higher in RA patients with combined anemia (ACD/IDA), but there was no significant difference in sRAGE levels between the subgroups based on hemoglobin levels. This result confirms the association of sRAGE with inflammation and also reflects the more severe condition of anemia of the chronic inflammatory process combined with pure iron deficiency (Figure 1).

It is noteworthy that the positive correlation of sRAGE with sTfR is observed independently of the sTfR-F index values and in the entire group of RA patients [33], confirming that the association between the cellular iron requirements and the chronic inflammatory process is essential.

It is known that IL-6 increases the expression of hepcidin, which regulates iron recycling and absorption, as it blocks iron release from body stores by downregulating ferroportin expression, leading to anemia [35]. Our results show that sRAGE is involved in anemia of the chronic inflammatory process, as positive correlations between sRAGE and prohepcidin and IL-6 (an inducer of hepcidin synthesis) were established only in the subgroup of patients with ACD (Table 1). Such an assumption has been reported by other authors who indicate sRAGE as a link between hypoferremia and inflammation, as it transduces signaling pathways of inflammation [31].

Autoantibodies are an immunological factor that significantly contributes to the etiology of RA. The significant positive correlations of sRAGE with specific immunologic indicators—RF and anti-CCP antibodies—were established only in patients with ACD and normal Hb levels. This result suggests that sRAGE reflects the autoimmune nature of the disease, but the association is lost in the presence of iron deficiency.

In patients with ACD/IDA, a positive correlation of sRAGE with DAS28 was observed, indicating an association between sRAGE and disease activity in the more severe combined type of anemia.

Previous studies provide evidence that RAGE is involved in bone remodeling. It appears that RANKL stimulates the expression of RAGE, which affects osteoclastogenesis, and knocking out RAGE attenuates RANKL-mediated osteoclast differentiation [36].

In our previous study, we reported a significant correlation between sRAGE and sRANKL in patients with RA [33]. The positive correlation between the two markers remains regardless of the hemoglobin levels and only in patients with ACD. This indicates that the association between the inflammatory changes (sRAGE) and osteoclast activation (sRANKL) is manifested only in patients with anemia of the chronic inflammatory process, but it loses its statistical significance in the complicated course of combined anemia (Table 1).

sRAGE and sRANKL are negatively correlated with serum iron only in the subgroup with combined anemia, where concomitant with bone resorption, an inflammatory process occurs that directs iron into the stores and causes hypoferremia (Table 1 and Table 2). These associations demonstrate that the decrease in serum iron in combined anemia may contribute to the promotion of bone erosions and the inflammatory process in RA.

Similar to sRAGE, sRANKL also showed sensitivity to the distribution of RA patients into subgroups according to the sTfR-F index, and its levels increase in the combination of ACD/IDA (Figure 1). The distribution of patients based on Hb levels does not result in a significant difference in sRANKL levels. The results indicate that sRANKL is a marker that is sensitive to the type of anemia.

We found that in RA, sTfR maintains a positive correlation not only with sRAGE but also with sRANKL, regardless of sTfR-F index and hemoglobin levels (Table 1 and Table 2). The results suggest significant interdependent mechanisms of adaptation of iron homeostasis, inflammation, and bone changes in the development of RA. These results show that regardless of the type of anemia, in RA patients, the cells protect their iron supply and form transferrin receptors in parallel with pro-inflammatory signals mediated by the expression of RAGE and RANKL.

The observed positive correlation between sRANKL and prohepcidin only in patients with low Hb levels and sTfR-F index < 1.5 confirms the association between sRANKL and iron homeostasis. We assume that sRANKL, like sRAGE, reflects iron deficiency, caused by increased IL-6 and prohepcidin (hepcidin precursor) in anemia of the chronic inflammatory process (Table 2).

We found statistically significant positive correlations of sRANKL with common diagnostic indicators such as DAS28, RF, and anti-CCP antibodies in RA patients [33]. When patients were separated based on the sTfR-F index, these correlations were observed only in the ACD subgroup. Taken together, these results indicate that sRANKL is an indicator of bone resorption, reflecting autoimmune status and disease activity in patients with anemia of the chronic inflammatory process but not in the complicated course of combined anemia (Table 2).

Fadda et al. found no significant correlation between OPG, RANKL, the OPG/RANKL ratio, and disease activity indicators such as DAS28 and ESR [37]. In our study, a correlation between sRANKL and OPG was also not found in RA patients [33]. But the comparison of subgroups based on the sTfR-F index showed that only in the group with anemia of inflammation, there was a positive correlation between sRANKL and OPG. This correlation suggests that as the potential for osteoclastogenesis increases (sRANKL), there is a parallel protection by osteoprotegerin in ACD (Table 2). In combined anemia, this correlation is absent, probably due to the presence of pure iron deficiency.

OPG likely shows sensitivity to the distribution of RA patients based on the sTfR-F index, as it tends to increase in the ACD subgroup (*p* = 0.053) compared to the combined anemia subgroup, as shown in Figure 1, but there is no significant difference in OPG levels between the subgroups based on Hb levels. When RA patients were distributed into subgroups based on the sTfR-F index and Hb levels, a positive correlation of OPG with sTfR was found in the subgroup with ACD. This suggests an adaptive mechanism of bone formation (OPG) in response to increased iron needs (sTfR) in anemia of the chronic inflammatory process. There is a negative correlation between OPG and serum iron in the subgroup with normal Hb levels and sTfR-F index < 1.5 (ACD). Such correlation indicates an adaptation of bone formation to anemia of the chronic inflammatory process, manifested by reduced iron delivery and preserved erythropoiesis (normal Hb levels) (Table 3).

OPG is expressed in response to an inflammatory process, as there is a positive correlation with CRP and IL-6 in the subgroup with ACD (Table 3), as well as in the entire group of RA patients [33]. OPG shows a relationship with the specific disease markers in RA only in anemia of chronic inflammation since there is a positive correlation of OPG with RF and DAS28 only in this patient subgroup (Table 3). Other authors have also reported a significant correlation between serum OPG and CRP and RF [38].

The positive correlation ferritin/OPG is observed in RA patients with normal Hb levels and sTfR-F index < 1.5 (ACD), as well as in the entire group of RA patients [33]. This suggests that osteoprotegerin expression increases under inflammatory conditions (elevated ferritin) as an adaptive mechanism.

Several potential limitations should be addressed in this pilot study. First, although the total sample size was not so limited, the number of the participants in some of the subgroups was small (especially the number of the subjects in the ACD/IDA cohort). Therefore, the results must be validated in larger subgroups for better statistical assessment. Second, because of the cross-sectional study design, we can only demonstrate mechanistic correlations but not causal relationships. Third, the pharmacological treatment of some patients could have influenced the obtained results [1].

## 4. Materials and Methods

### 4.1. Patient and Subgroup Characteristics

This study included 16 male and 98 female rheumatoid arthritis (RA) patients recruited from the Rheumatology Department of the University hospitals at the Medical University of Plovdiv. The description of the study participants and their pharmacological treatment was reported in our previous studies [33,34]. Patients fulfilled the 2010 EULAR (European Alliance of Associations for Rheumatology) classification criteria for RA. Written informed consent was obtained from all participants according to the Declaration of Helsinki after approval by the Ethics Committee of the Medical University of Plovdiv. The subject inclusion criteria were women and men aged between 18 and 80 years who fulfilled the EULAR criteria for RA. The subject exclusion criteria were patients with psoriatic arthritis, patients with systemic lupus erythematosus, patients with RA having renal or hepatic impairment, patients with hemolytic anemia, patients who had blood transfusions within the past three months, cancer patients currently receiving chemotherapy, and patients currently taking iron supplements.

In the present study, RA patients were distributed into subgroups based on sTfR-F index and hemoglobin levels. Subjects with sTfR-F index value < 1.5 were classified as having anemia of chronic disease—ACD—and patients with sTfR-F index value > 1.5 were classified as having anemia of chronic disease with coexistent iron deficiency anemia—ACD/IDA [39,40,41]. The subgroup with ACD consisted of 100 patients, and the subgroup with ACD/IDA consisted of 14 patients. The World Health Organization (WHO) definitions of anemia were used as a hemoglobin threshold of <12 g/dL in women and <13 g/dL in men. The first subgroup was composed of 79 non-anemic patients, and the second subgroup was made up of 35 patients who fulfilled the criteria for anemia.

The main demographic and clinical characteristics of the study groups are summarized in Table 4.

### 4.2. Laboratory Analyses

The concentrations of ferritin, ng/mL; sTfR, μg/mL; C-reactive protein (CRP), μg/mL; interleukin 6 (IL-6), pg/mL; sRAGE, pg/mL; sRANKL, pmol/L; and OPG, pmol/L, in serum samples were determined using the ELISA method (BioVendor—Laboratorni medicina, Brno, Czech Republic). Serum prohepcidin levels, ng/mL, were measured with ELISA kits (DRG Instruments, GmbH, Marburg, Germany); rheumatoid factor (RF), U/mL, was measured with ELISA kits (Nova Tec Immundiagnostica, GmbH, Dietzenbach, Germany); and anti-cyclic citrullinated peptide (antiCCP) antibodies, U/mL, were measured with ELISA kits (Eurodiagnostica, Malmö, Sweden). All ELISA tests were performed following the procedures from the manufacturer’s instructions, and the absorbance of the samples was measured with an ELISA reader HumaReader HS, HUMAN (Wiesbaden, Germany). Serum iron, µmol/L, was determined with the colorimetric method and TPTZ [2,4,6–three-(2-pyridyl)-5-triazine] as a chromogen.

### 4.3. Statistical Analyses

The software IBM SPSS version 17.0 was used to conduct the statistical analysis. The Kolmogorov–Smirnov test was used to assess the distribution of the samples, and continuous variables were presented as mean ± SD or as median (25th percentile–75th percentile). The differences between two groups were analyzed with Student’s *t*-test for groups with Gaussian distribution and with the Mann–Whitney U test for groups with non-Gaussian distribution. Statistical relationships between two variables were evaluated using Pearson’s or Spearman’s correlation coefficient depending on their distribution; *p* < 0.05 was considered as statistically significant.

## 5. Conclusions

It is notable that soluble receptors are important participants in anemia of the chronic inflammatory process, as correlation links in the sRAGE–sRANKL–OPG axis are found only in this subgroup of patients. This likely indicates the presence of relationships mediated by additional factors in the complicated course of combined anemia, which require further investigation. The decrease in serum iron content in combined anemia may contribute to the promotion of bone erosions and inflammation in RA assessed by sRAGE and sRANKL.

The results obtained indicate that there is a difference in both the mean values and the correlation links of sRAGE, sRANKL, and OPG between the subgroups of patients with functional and combined iron deficiency. Therefore, the investigated soluble receptors involved in the pathogenesis of the disease are specific reliable biomarkers sensitive to the type of anemia, assessed by the sTfR-F index. The level of soluble receptors likely reflects the more severe condition of anemia of chronic inflammation combined with pure iron deficiency in RA.

## Figures and Tables

**Table 1:** Correlations of sRAGE in the subgroups of RA patients according to the level of the sTfR-F index (ACD and ACD/IDA) and Hb (no anemia and anemia).

Parameters	r	*p*	r	*p*
	ACD		ACD/IDA	
**sRAGE/sTfR**	0.356	<0.0001 *	0.714	0.004 *
**sRAGE/serum iron**	−0.211	NS	−0.976	0.005 *
**sRAGE/prohepcidin**	0.260	0.009 *	0.431	NS
**sRAGE/sRANKL**	0.600	<0.0001 *	0.420	NS
**sRAGE/CRP**	0.199	0.048 *	−0.215	NS
**sRAGE/DAS28**	0.049	NS	0.566	0.035 *
**sRAGE/IL-6**	0.377	0.005 *	−0.304	NS
**sRAGE/RF**	0.302	0.003 *	−0.054	NS
**sRAGE/AntiCCP antibodies**	0.471	<0.0001 *	−0.064	NS
	**No anemia**		**Anemia**	
**sRAGE/sTfR**	0.519	<0.0001 *	0.322	0.059
**sRAGE/prohepcidin**	0.272	0.015 *	0.332	0.052
**sRAGE/sRANKL**	0.740	<0.0001 *	0.495	0.003 *
**sRAGE/IL-6**	0.246	NS	0.512	0.043 *
**sRAGE/CRP**	0.229	0.042 *	0.075	NS
**sRAGE/RF**	0.396	0.001 *	0.209	NS
**sRAGE/AntiCCP antibodies**	0.460	0.001 *	0.379	NS

* statistical significance.

**Table 2:** Correlations of sRANKL in the subgroups of RA patients according to the level of the sTfR-F index (ACD and ACD/IDA) and Hb (No anemia and Anemia).

Parameters	r	*p*	r	*p*
	ACD		ACD/IDA	
**sRANKL/sRAGE**	0.600	<0.0001 *	0.420	NS
**sRANKL/sTfR**	0.527	<0.0001 *	0.657	0.011 *
**sRANKL/serum iron**	−0.213	NS	−0.927	0.023 *
**sRANKL/ferritin**	0.174	NS	0.565	0.035 *
**sRANKL/prohepcidin**	0.298	0.003 *	−0.138	NS
**sRANKL/CRP**	0.457	<0.0001 *	0.190	NS
**sRANKL/OPG**	0.238	0.017 *	−0.093	NS
**sRANKL/IL-6**	0.355	0.009 *	−0.508	NS
**sRANKL/DAS28**	0.318	0.001 *	0.072	NS
**sRANKL/RF**	0.403	<0.0001 *	0.396	NS
**sRANKL/AntiCCP antibodies**	0.545	<0.0001 *	0.352	NS
	**No anemia**		**Anemia**	
**sRANKL/sRAGE**	0.740	<0.0001 *	0.495	0.003 *
**sRANKL/sTfR**	0.680	<0.0001 *	0.586	<0.0001 *
**sRANKL/prohepcidin**	0.199	NS	0.538	0.001 *
**sRANKL/CRP**	0.354	0.001 *	0.414	0.013 *
**sRANKL/IL-6**	0.299	0.051	0.364	NS
**sRANKL/DAS28**	0.207	0.067	0.460	0.005 *
**sRANKL/RF**	0.361	0.002 *	0.587	<0.0001 *
**sRANKL/AntiCCP antibodies**	0.459	0.001 *	0.585	0.005 *

* statistical significance.

**Table 3:** Correlations of OPG in the subgroups of RA patients according to the level of the sTfR-F index (ACD and ACD/IDA) and Hb (No anemia and Anemia).

Parameters	r	*p*	r	*p*
	ACD		ACD/IDA	
**OPG** **/ferritin**	0.224	0.025 *	0.281	NS
**OPG** **/sTfR**	0.226	0.024 *	0.243	NS
**OPG** **/serum iron**	−0.380	0.005 *	0.048	NS
**OPG** **/sRANKL**	0.238	0.017 *	−0.093	NS
**OPG** **/CRP**	0.238	0.017 *	0.180	NS
**OPG** **/IL-6**	0.269	0.049 *	0.714	NS
**OPG** **/RF**	0.238	0.022 *	−0.464	NS
**OPG** **/DAS28**	0.215	0.032 *	−0.377	NS
	**No anemia**		**Anemia**	
**OPG/ferritin**	0.312	0.005 *	0.178	NS
**OPG/** **serum iron**	−0.398	0.010 *	0.006	NS
**OPG/IL-6**	0.215	NS	0.542	0.030 *
**OPG/RF**	0.026	NS	0.350	0.042 *

* statistical significance.

**Table 4:** Demographic and clinical characteristics of the study groups.

Characteristics	ACD, n = 100	ACD/IDA, n = 14	No Anemia, n = 79	Anemia, n = 35
**Mean age, years**	58 ± 10	54 ± 8	58 ± 9	57 ± 12
**Sex (M/F)**	15/85	1/13	13/66	3/32
**DAS28**	5.7 ± 1.1	5.8 ± 1.0	5.4 ± 1.1	6.3 ± 1.2
**RF, U/mL**	51 (18–190)	90 (50–330)	56 (20–182)	61 (18–209)
**AntiCCP antibodies, U/mL**	148 (51–246)	129 (109–911)	127 (50–235)	181 (89–736)

Note: Data are presented as mean ± SD or median values (25th–75th percentile); rheumatoid factor (RF), anti-cyclic citrullinated peptide (antiCCP) antibodies.

## Data Availability

The data presented in this study are available upon reasonable request from the corresponding author.
